# *NFIX* as a Master Regulator for Lung Cancer Progression

**DOI:** 10.3389/fphar.2017.00540

**Published:** 2017-08-21

**Authors:** Nor I. A. Rahman, Nor A. Abdul Murad, Mohammad M. Mollah, Rahman Jamal, Roslan Harun

**Affiliations:** ^1^UKM Medical Molecular Biology Institute (UMBI), National University of Malaysia Kuala Lumpur, Malaysia; ^2^Department of Paediatrics, Faculty of Medicine, National University of Malaysia Kuala Lumpur, Malaysia; ^3^KPJ Ampang Puteri Specialist Hospital Ampang, Malaysia

**Keywords:** master regulator, transcription factor, transcriptional regulator, lung cancer, *NFIX*, metastasis

## Abstract

About 40% of lung cancer cases globally are diagnosed at the advanced stage. Lung cancer has a high mortality and overall survival in stage I disease is only 70%. This study was aimed at finding a candidate of transcription regulator that initiates the mechanism for metastasis by integrating computational and functional studies. The genes involved in lung cancer were retrieved using *in silico* software. 10 kb promoter sequences upstream were scanned for the master regulator. Transient transfection of shRNA NFIXs were conducted against A549 and NCI-H1299 cell lines. qRT-PCR and functional assays for cell proliferation, migration and invasion were carried out to validate the involvement of *NFIX* in metastasis. Genome-wide gene expression microarray using a HumanHT-12v4.0 Expression BeadChip Kit was performed to identify differentially expressed genes and construct a new regulatory network. The *in silico* analysis identified *NFIX* as a master regulator and is strongly associated with 17 genes involved in the migration and invasion pathways including *IL6ST*, *TIMP1* and *ITGB1*. Silencing of *NFIX* showed reduced expression of *IL6ST*, *TIMP1* and *ITGB1* as well as the cellular proliferation, migration and invasion processes. The data was integrated with the *in silico* analyses to find the differentially expressed genes. Microarray analysis showed that 18 genes were expressed differentially in both cell lines after statistical analyses integration between *t*-test, LIMMA and ANOVA with Benjamini-Hochberg adjustment at *p*-value < 0.05. A transcriptional regulatory network was created using all 18 genes, the existing regulated genes including the new genes *PTCH1*, *NFAT5* and *GGCX* that were found highly associated with *NFIX*, the master regulator of metastasis. This study suggests that *NFIX* is a promising target for therapeutic intervention that is expected to inhibit metastatic recurrence and improve survival rate.

## Introduction

The 5-year survival rate of patients with stage 1A and 1B non–small cell lung cancer (NSCLC) is about 49% and 45% respectively (American Cancer Society, 2015). The poor prognosis in lung cancer suggests that some cells in the primary tumours are programmed to metastasize (Quail and Joyce, 2013; Seyfried and Huysentruyt, 2013). Lung cancer is usually asymptomatic in the early stage and typically symptoms arise when the tumor has metastasized (Slatore et al., 2011). Besides drug resistance, metastasis is also a major cause in cancer recurrence and a big concern in treatment effectiveness (Tang et al., 2013). Therefore, it is crucial to determine the regulatory network that controls the metastatic genes in lung cancer to improve prognosis and increase the survival rate of patients. These genes are not well defined because of the gene transcription changes at every stage of cancer (Shibayama et al., 2011). However, identification of the transcription factors (TFs) that regulate the metastasis mechanisms could be a novel approach for reducing metastasis in lung cancer.

Previous studies of colorectal cancer have indicated that distinct gene expression profiles are associated with disease progression and metastatic recurrence (Shibayama et al., 2011). Metastasis is a multi-step process where tumor cells interact with their microenvironment (Chiang and Massagué, 2008). Expression of the metastatic signature has been directly associated with poor prognosis in most cancers, including colorectal and breast cancers (Hanahan and Weinberg, 2000). For example, expression of the *ELAC1* gene in colorectal cancer is essential for inducing tumor development, cell growth and disruption of apoptosis pathways (Kleivi et al., 2007). *MTA1* gene is involved in the progression and metastasis of breast cancer (Gururaj et al., 2006). Overexpression of *MTA1* also promotes the transcription of oncogenes (Gururaj et al., 2006; Pakala et al., 2013).

Patients with metastatic gene expression shows poor prognosis compared to patients with no metastatic gene signatures (Ramaswamy et al., 2003). The authors suggested that rare cells within a primary tumor have the metastatic phenotype, where they can migrate and invade other cells (Ramaswamy et al., 2003). Although more than 90% of metastasis signatures have been discovered, the metastatic mechanisms of these signatures are largely unknown (Sleeman and Steeg, 2010). Gene expression profiling for identification of molecular signatures results in extensive data on differentially expressed genes (Rohrbeck et al., 2008). The gene expression data provides an insight into every step of lung carcinogenesis according to the different tumor morphology (Borczuk et al., 2003). Using the bioinformatics software, a molecular network on metastasis can be constructed and the TFs that control the metastasis process can be identified.

Predicting the specific binding of TFs to a DNA sequence is the main key in constructing a specific transcriptional regulation network which leads to the metastatic process. To facilitate understanding of the metastatic mechanisms, the TFs need to be identified first; hence the regulatory network can be constructed. These TFs could potentially be targeted to control lung cancer metastasis, thus could improve the survival and prognosis of patients with lung cancer.

## Materials and Methods

### Identification of Candidate Metastatic Lung Cancer Genes

Nine lung cancer datasets were selected from the Oncomine database^1^: Bhattacharjee, Beer, Bild, Hou, Lee, The Cancer Genome Atlas (TCGA), TCGA2, Tomida and Lung. These datasets are based on the original microarray analyses on lung cancer by various researchers that published and pooled together in data-mining platform of Oncomine for easy discovery. The difference between both TCGAs is that the second dataset (TCGA2) is actually containing the data from new samples profiled since the first published. Genes related to non–small cell primary lung cancer adenocarcinoma, the 5-year survival rate and stage I and II pathology subtypes were retrieved using the given filters. All datasets were compared except for the outliers, and the cut-off point was set at *p*-value < 0.01. FunDO is a functional disease ontology annotation database (django.nubic.northwestern.edu/fundo/) lists the genes involved in various cancers (Osborne et al., 2009). The common genes involved in cancers and lung cancer obtained were classified according to their biological processes and molecular functions (cell–cell adhesion, cell migration, cell motion, cell death, apoptosis, programmed cell death, regulation of locomotion, cell migration, cell cycle, cell division, localization of cell, cellular differentiation, cell viability, inflammation, regulation of transcription, angiogenesis and oncogenic signaling pathway) were using DAVID Bioinformatics Database Version 6.7 (Huang et al., 2008). Integrated genes from DAVID analysis underwent further filtration by Pathway Studio analysis with the cut-off point at *p*-value < 0.01. Overlapping genes were selected using GeneVenn (Pirooznia et al., 2007).

### Identification of the Master Regulator of Metastatic Lung Cancer

10kb promoter sequences upstream from the transcription start site (TSS) of the final candidate genes were retrieved from the Eukaryotic Promoter Database (EPD^2^) and the National Centre for Biotechnology Information (NCBI^3^) for TF recognition in FASTA format. MATCH, a TRANSFAC^®^ gene regulation database program, was used for predicting putative transcription factor binding sites (TFBS) in DNA sequences. TFBS library in the TRANSFAC^®^ database was used to construct specific binding site weight matrices in TFBS prediction. A maximum core dissimilarity cut-off value of 15% (85% core similarity) was chosen for each matrix. This parameter shows the similarity of a sequence and the weight matrix used by the system to report the TF as a true binding site. PROMO version 3.0.2^4^ scans and identifies TFBS in promoter regions by weight matrix search (Farré et al., 2003; Messeguer et al., 2012). Factors predicted within a dissimilarity margin less or equal to 15%.

### Identification of Specific Genes and Their Master Regulator

Transcription factor binding sites derived from both MATCH and PROMO were integrated to identify the common TFs of the regulated genes. To obtain novel findings, we constructed a network that includes all regulated genes and the predicted TFs using Pathway Studio and String 2.0. One of the direct networks was selected based on *in silico* prediction results as shown in Section “Results.”

### Lentiviral Vector and Cell Lines Used

The lentiviral vector with NFIX plasmids (shRNA) was purchased from Thermo Scientific Open Biosystems (BD Biosciences, United States). Plasmids were cultured in Luria broth with ampicillin (AMRESCO, United States) and DNA was extracted using Qiagen purification kit (Germany) for transient transfection. Two human lung cancer cell lines, A549 and NCI-H1299 were purchased from American Type Culture Collection (United States). A549 cells, from a human lung carcinoma cell, maintained in Kaighn’s modification of Ham’s F-12 (F-12K) medium (Thermo Fisher Scientific, United States). NCI-H1299 cells, also derived from a human lung carcinoma and metastatic site lymph nodes, maintained in Roswell Park Memorial Institute1640 (RPMI1640) medium (Thermo Fisher Scientific, United States). Both medium were supplemented with 10% fetal bovine serum (Thermo Fisher Scientific, United States).

### Transient Transfection of NFIX shRNA

4 × 10^4^ cells were cultured with serum-free medium without antibiotics in a 6-well culture plate until 60–70% confluent. One microgram of shRNA DNA was transiently transfected into the cells using TurboFECT (Thermo Fisher Scientific, United States). Three *NFIX* shRNAs, *GAPDH* shRNA (endogenous control) and non-silencing plasmid were used to study the effects of *NFIX* loss of function. Transfection efficiency was measured using the fluorescent imaging software NIS-Elements (Nikon Instrument Inc., United States) standardized in all materials and methods. The experiment was performed in triplicate.

### Gene Expression Analyses Using Quantitative PCR (qPCR)

Total RNA was isolated using a NucleoSpin^®^ RNA II kit (Macherey-Nagel, United States) according to the manufacturer’s protocol and purity was determined using NanoDrop (Thermo Fisher Scientific, United States). Following the High-Capacity RNA-to-cDNA^TM^ kit (Thermo Fisher Scientific, United States) protocol, 1 μg RNA was converted to cDNA. Gene expression analyses were conducted using TaqManFast Advanced Master Mix (Thermo Fisher Scientific) and TaqMan^®^ GeneExpression Assay (Thermo Fisher Scientific, United States) using 7500 Fast Real-Time PCR machine (Thermo Fisher Scientific, United States). The experiment was conducted in triplicate.

### Cell Proliferation Assay

1 × 10^4^ cells were cultured in a 96-well culture plate and transfected with 1 μg of each *NFIX* shRNA in the respective wells and incubated for 48 h. CellTiter 96^®^AQueous One Solution Cell Proliferation (MTS) (20 μl) (Promega, United States) was added to each well and incubated for 4 h before readings were obtained using Varioskan Flash (Thermo Fisher Scientific) at 490 nm. The experiment was conducted in triplicate.

### Cell Migration and Cell Invasion Assays

The QCM^TM^ Chemotaxis (3 μm) migration kit (Chemicon, United States) and QCM^TM^ 24-well Fluorimetric Cell Invasion assay kit (Millipore, United States) were used to measure the migration and invasion activities of the transfected cell lines. The membrane of the ECMatrix^TM^ in the invasion kit only allows invasive cells to migrate toward the underside of the insert filter into the bottom well. In total, 1 × 10^6^ cells were harvested in 1ml serum-free medium and 250 μl cells were pipetted into the insert in triplicate for each treatment. About 400 and 500 μl complete culture medium was filled in the bottom of the well before soaking the insert. After 48 h incubation, the migrated and invaded cells were detached using cell detachment solution and dyed with cyQUANT^®^ GR and 4X Lysis Buffer in a ratio of 1:75. The migrated and invaded cells at the bottom of wells were measured using VarioskanFlash (Thermo Fisher Scientific, United States) at 480/520 nm. The experiment was conducted in triplicate.

### Gene Expression Microarray

Transfected cells were collected and RNA was extracted using NucleoSpin^®^ RNA II (Macherey-Nagel, Germany) and diluted with RNase-free water to a final concentration of 150 ng/μl. RNA concentration and purity were measured using NanoDrop (Thermo Fisher Scientific, United States) and Bioanalyzer 2100 RNA 6000 kit (Agilent Technologies, United States). The RNA integrity number (RIN) must be >7 for microarray experiments. TargetAmp^TM^ Nano Labelling Kit for Illumina^®^ Expression BeadChip^®^(Epicentre Biotechnologies, United States) was used for RNA labeling. The cDNA purification step was carried out using a MinElute Reaction Clean-up (50) kit (Qiagen, Germany). A HumanHT-12v4.0 BeadChip expression kit was used to hybridize the purified cDNA samples. A gene expression direct hybridisation assay system from Illumina was used to process the BeadChip. Cy3-streptavidin (Thermo Fisher Scientific, United States) was introduced as biotin to the analytical probes in the hybridized BeadChip. The iScan Image BeadChip software system was used to scan the BeadChip. Genome Studio, Partek and R software were utilized to analyze the microarray data.

## Results

### *In Silico* Analyses for Identifying Master Regulator and the Candidate Genes

**FIGURE 1:**
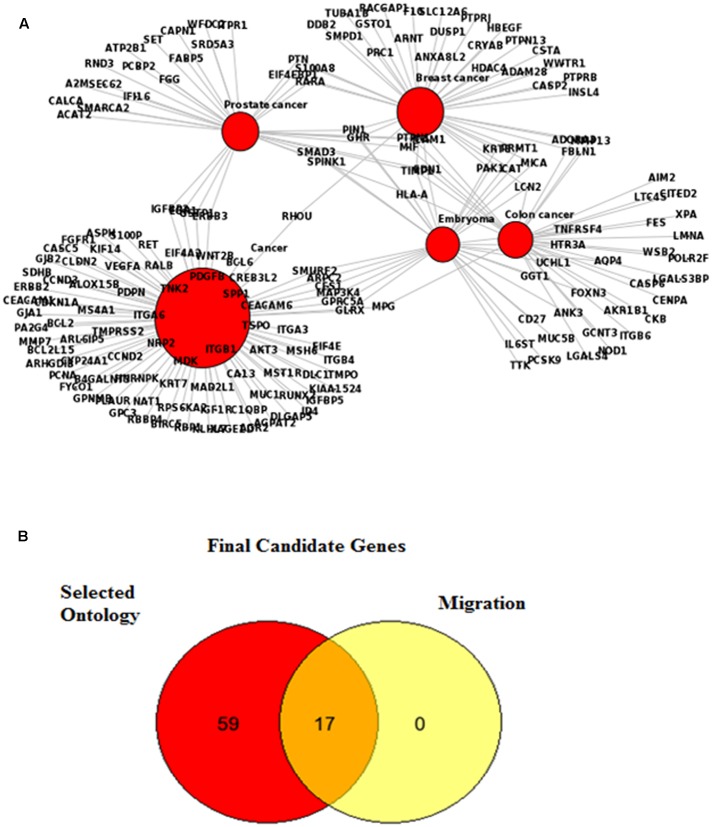
FunDO analysis revealed the molecular networks for various cancers. **(A)** Genes such as *SMAD3*, *HLA-A*, *S100A8* and *RHOU* were common in prostate, breast and colon cancers. **(B)** Venn diagram showing the 17 candidate genes involved in migration as well as other common ontology in cancers.

**FIGURE 2:**
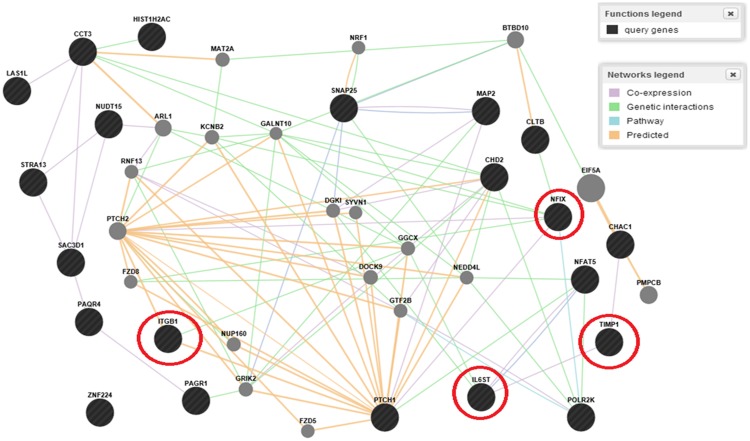
Circled are the known regulated genes and predicted master regulator *NFIX* as a final direct network that underwent the validation based on *in-silico* prediction results.

Nine datasets were obtained from the Oncomine database and 3561 genes were selected based on *p*-value < 0.01 after filtration for the criteria of lung adenocarcinoma, 5-year survival rate and stage I and II pathological subtypes. Early stages of cancer were used to find the genes commonly appear compared to the late stages as we believed they are aiding to metastasis in the micrometastasis stage. The FunDO results were presented in two data sets: one is showing the gene list in lung cancer and the other one is containing the gene list for various cancers such as colorectal, breast, embryoma and prostate. **Tables 1**, **2** show the gene list involved in lung cancer and various cancers respectively. **Figure 1A** shows the network involved in various diseases from 549 up-regulated and 674 down-regulated genes. About 18 genes are involved in lung cancer regulation and 88 genes are shared by various cancers based on the gene network that is automatically generated by FunDO analysis using the algorithm set up with Unified Medical Language System (UMLS) MetaMap Transfer tool (MMTx) to discover gene-disease relationship from the GeneRIF database. All 106 genes were enriched and classified by selected ontology using DAVID and Pathway Studio. All genes involved in the migration pathway were selected as final candidate genes. **Figure 1B** shows all candidate of transcription regulator and **Table 3** shows the final gene list commonly involved in lung cancer with their respected direction of regulation in lung cancer including *ALOX15B*, *CALCA*, *CEACAM1*, *DLC1*, *ICAM1*, *IL6ST*, *ITGB1*, *RET*, *S100P*, *VEGFA*, *BCL2*, *IGF1R*, *IGFBP5*, *ITGA6*, *NRP2*, *PDGFB* and *PDPN*. **Table 4** shows the final candidate of TFs involved in lung cancer metastasis regulation, i.e., *AP1, SP1, GR, FOXJ1, GATA6, NF1, GATA5, TTF1(NKX2.1), ATF2, C/EBP*, *C/EBPG*, *C-JUN*, myogenin*/NF1*, *HNF3B*, *RXR:RAR*, *CTF1*, *FOXL1*, *TAX/CREB* and *NF1A/NFIX.* We selected nuclear factor I X (*NFIX*) as the candidate master regulator as it is a family of closely related TFs that constitutively bind as dimers to specific DNA sequences with high affinity. We chose *NFIX* instead of another TFs since it has higher occurrence. *NFIX* is also less studied especially in lung cancer. Furthermore, *NFIX* has a high occurrence in 10 kb upstream of the promoter sequences of the candidate genes. We constructed a network consist of all our known regulated genes and the predicted TFs using Pathway Studio and String 2.0, however, we only pick one direct network based on *in silico* prediction results as shown in **Figure 2**. The signaling network of *IL6ST* was evaluated and *TIMP1* was established as the gene that is co-expressed in the migration program. In addition, *ITGB1* was selected as the final candidate gene to complete the network as it is involved in the migration and invasion pathways as determined in the previous analyses.

**Table 1:** Functional disease ontology annotation database (FunDO) identified gene involved in lung cancer regulation.

Rank	Gene	Entrez ID
1	*GHR*	2690
2	*CSTA*	1475
3	*TIMP1*	7076
4	*ADAM8*	101
5	*P2RY6*	5031
6	*FGFR1OP*	11116
7	*LGALS3BP*	3959
8	*KLRB1*	3820
9	*CCNB2*	9133
10	*ADAM28*	10863
11	*KRT8*	3856
12	*UCHL1*	7345
13	*XPA*	7507
14	*IL6ST*	3572
15	*ICAM1*	3383
16	*MIF*	4282
17	*CALCA*	796
18	*FEN1*	2237


**Table 2:** Functional disease ontology annotation database (FunDO) identified genes involved in various cancer networks.

Rank	Gene	Entrez ID	Rank	Gene	Entrez ID	Rank	Gene	Entrez ID
1	*CDKN1A*	1026	30	*DLC1*	10395	59	*WNT2B*	7482
2	*NAT1*	9	31	*GPC3*	2719	60	*RBP1*	5947
3	*ERBB2*	2064	32	*KLHL7*	5597	61	*ID4*	3400
4	*ERBB3*	2065	33	*VEGFA*	7422	62	*BIRC5*	332
5	*PDGFB*	5155	34	*GLRX*	2745	63	*CA13*	377677
6	*SPP1*	6696	35	*TSPO*	706	64	*RET*	5979
7	*GPRC5A*	9052	36	*CIQBP*	708	65	*RUNX2*	860
8	*CES1*	1066	37	*KIFI4*	9928	66	*AGPAT2*	10555
9	*RPS6KA2*	6196	38	*PLAUR*	5329	67	*CLDN2*	9075
10	*CYP24A1*	1591	39	*FGFR1*	2260	68	*ARPC2*	10109
11	*DLGAP5*	9787	40	*GPNMB*	10457	69	*CCND2*	894
12	*MST1R*	4486	41	*GSTP1*	2950	70	*CCND3*	896
13	*ITGA6*	3655	42	*MSH6*	2956	71	*B4GALNT3*	283358
14	*CEACAM6*	4680	43	*SMURF2*	64750	72	*MMP7*	4316
15	*FYCO1*	79443	44	*SDHB*	63905	73	*ARHGDIB*	397
16	*BCL2*	596	45	*ALOX15B*	247	74	*IGF1R*	3480
17	*ITGA3*	3675	46	*CASC5*	57082	75	*IGFBP2*	3485
18	*BCL6*	604	47	*CREB3L2*	64764	76	*IGFBP5*	3488
19	*MDK*	4192	48	*MPG*	4350	77	*MS4A1*	931
20	*ITGB1*	3688	49	*RALB*	5899	78	*EGR1*	1958
21	*ITGB4*	3691	50	*KRT7*	3855	79	*PA2G4*	5036
22	*RHOU*	58480	51	*AKT3*	10000	80	*EIF4A2*	1974
23	*HNRNPK*	3190	52	*BCL2L15*	440603	81	*EIF4E*	1977
24	*MAP3K4*	4216	53	*XAGEID*	9503	82	*ASPH*	444
25	*CEACAM1*	634	54	*PDPN*	10630	83	*TMPO*	7112
26	*NRP2*	8828	55	*RBBP4*	5928	84	*TMPRSS2*	7113
27	*GJA1*	2697	56	*KIAA1524*	57650	85	*TNK2*	10188
28	*S100P*	6286	57	*ARL6IP5*	10550	86	*MUC1*	4582
29	*GJB2*	2706	58	*AGR2*	10551	87	*MAD2L1*	4085
						88	*PCNA*	5111


**Table 3:** Final gene list prior to transcription factors (TFs) scanning by National Centre for Biotechnology Information (NCBI), Eukaryotic Promoter Database (EPD) and PROMO.

No.	Expression in Lung Cancer	Gene
1	UP	*ALOX15B*
2	UP	*CALCA*
3	UP	*CEACAM1*
4	UP	*DLC1*
5	UP	*ICAM1*
6	UP	*IL6ST*
7	UP	*ITGB1*
8	UP	*RET*
9	UP	*S100P*
10	UP	*VEGFA*
11	DOWN	*BCL2*
12	DOWN	*IGF1R*
13	DOWN	*IGFBP5*
14	DOWN	*ITGA6*
15	DOWN	*NRP2*
16	DOWN	*PDGFB*
17	DOWN	*PDPN*


**Table 4:** Transcription factor binding sites (TFBS) of up-regulated and down-regulated genes that occurred in MATCH and PROMO, scanned up to 10 kb upstream of the promoter sequences from the transcription start site (TSS).

Rank of occurrence	TFBS
1	*AP1*
1	*SP1*
2	*GR*
3	*FOXJ1*
4	*GATA5*
5	Myogenin*/NF1*
6	*GATA6*
7	*TTF1* (*NKX2.1*)
8	*ATF2*
9	*C/EBP*
9	*C/EBPG*
9	*C-JUN*
9	*NF1*
10	*C/EBPA*
11	*HNF3B*
11	*RXR:RAR*
12	*CTF1*
12	*FOXL1*
13	*NF1A/NFIX*
13	*TAX/CREB*


### Effect of *NFIX* Silencing on the Regulated Genes *IL6ST, TIMP1* and *ITGB1*

**FIGURE 3:**
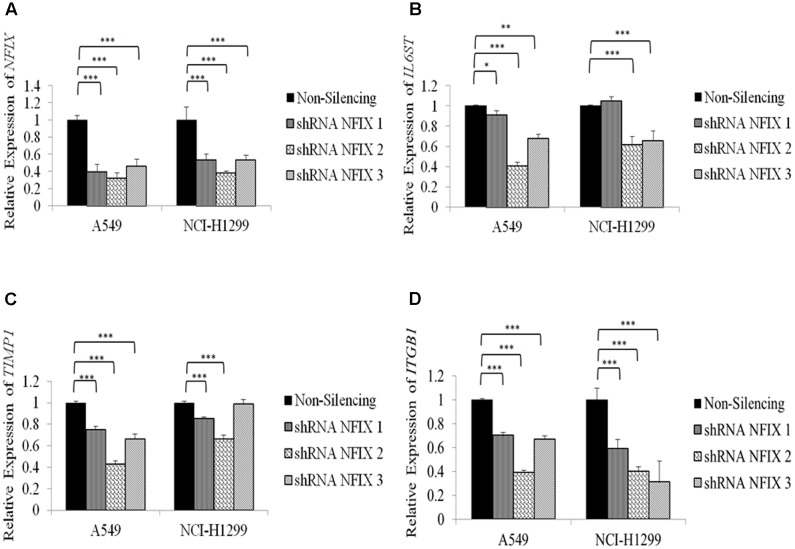
A549 and NCI-H1299 cell lines transfected for 48 h with *NFIX* shRNAs had reduced *NFIX* expression. **(A)** Relative expression of *NFIX* after 48 h *NFIX* shRNAs transfection. **(B)** Transfection of *NFIX* shRNAs reduced *IL6ST* gene expression in both cell lines. **(C)** Transfection with NFIX_2shRNA showed the most significant reduction of TIMP1gene expression. **(D)** Transfection of *NFIX* shRNAs significantly reduced *ITGB1* gene expression in both cell lines. ^∗^*p* < 0.01;^∗∗^ 0.01 < *p* < 0.05; ^∗∗∗^*p* < 0.05.

Relative expression of *NFIX* in A549 cells was decreased significantly with *p*-value < 5.0 × 10^-4^ for each construct 48h post-transfection. NCI-H1299 cells transfected with *NFIX* shRNA also showed significant reduction of *NFIX* gene expression. NFIX_2 shRNA caused the most significant reduction of *IL6ST* expression in A549 and NCI-H1299 cells (*p*-value = 1.2 × 10^-4^ and 7.99 × 10^-5^, respectively). NFIX_2 shRNA caused the most significant reduction of *TIMP1* expression in A549 cells (*p*-value = 2.71 × 10^-8^) and NCI-H1299 cells (*p*-value = 4.98 × 10^-7^). All *NFIX* shRNAs caused significant reduction in *ITGB1* expression in both A549 and NCI-H1299 cells. **Figure 3** shows *NFIX*, *IL6ST*, *TIMP1* and *ITGB1* expression in both cell lines after 48 h transfection with *NFIX* shRNAs.

### *NFIX* Reduced Cellular Viability and Proliferation

**FIGURE 4:**
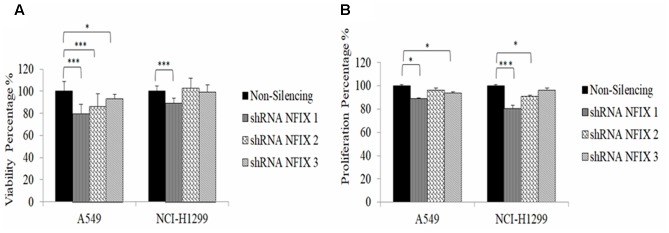
Cellular viability and proliferation activities were reduced in both cell lines at 48 h post-transfection with NFIX shRNAs. **(A)** A549 cell viability was decreased following transfection with all NFIX shRNAs. **(B)** NCI-H1299 cell viability was reduced only by NFIX_1shRNA. ^∗^*p* < 0.01; ^∗∗∗^*p* < 0.05.

NFIX_1 shRNA transfection reduced cellular viability significantly in both cell lines (A549: *p*-value = 5.87 × 10^-5^; NCI-H1299: *p*-value = 2.4 × 10^-4^) 48h post-transfection. Cellular proliferation was reduced significantly at 48 h post-transfection with NFIX_1 shRNA in both A549 and NCI-H1299 (*p*-value = 0.05 and 0.01, respectively). **Figure 4** shows the reduction in cellular viability and proliferation in both cells 48 h after *NFIX* shRNAs transfection.

### *NFIX* Decreased Cellular Migration and Invasion

**FIGURE 5:**
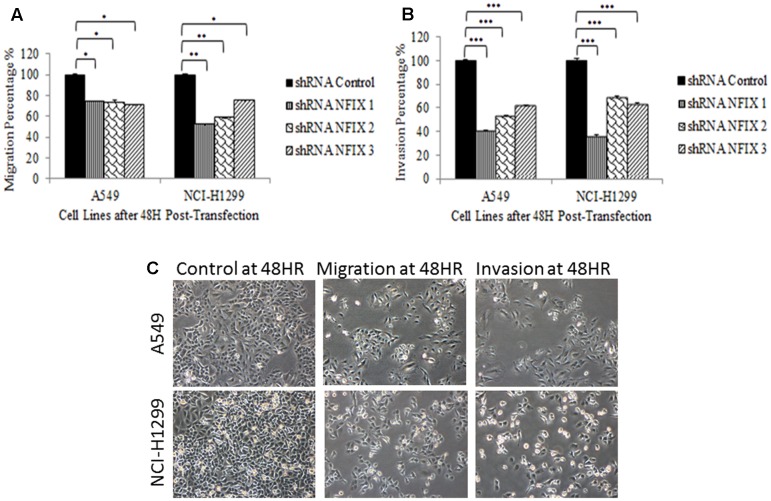
Reduction in cellular migration and invasion activities at 48 h post-transfection. **(A)** All *NFIX* shRNAs decreased migration in both cell lines. **(B)** All *NFIX* shRNAs significantly reduced invasion in both cell lines. **(C)** Transwell migration and invasion assays compared with each control cell lines. (^∗^*p* < 0.01; ^∗∗^0.01 < *p* < 0.05; ^∗∗∗^p < 0.05).

Transfection of all *NFIX* shRNAs significantly reduced the migration and invasion activities in both cell lines at 48h post-transfection. Transfection of NFIX_1 shRNA showed the most significant reduction in both cell lines after 48h (A549: *p*-value = 7.9 × 10^-3^; NCI-H1299: *p*-value = 7.3 × 10^-3^). This observation suggests that *NFIX* may play an important role in lung cancer metastasis. **Figure 5** shows that *NFIX* shRNAs transfection reduced migration and invasion activities in both cell lines.

### Microarray Analysis of Master Regulator and Regulatory Network in Lung Cancer

**FIGURE 6:**
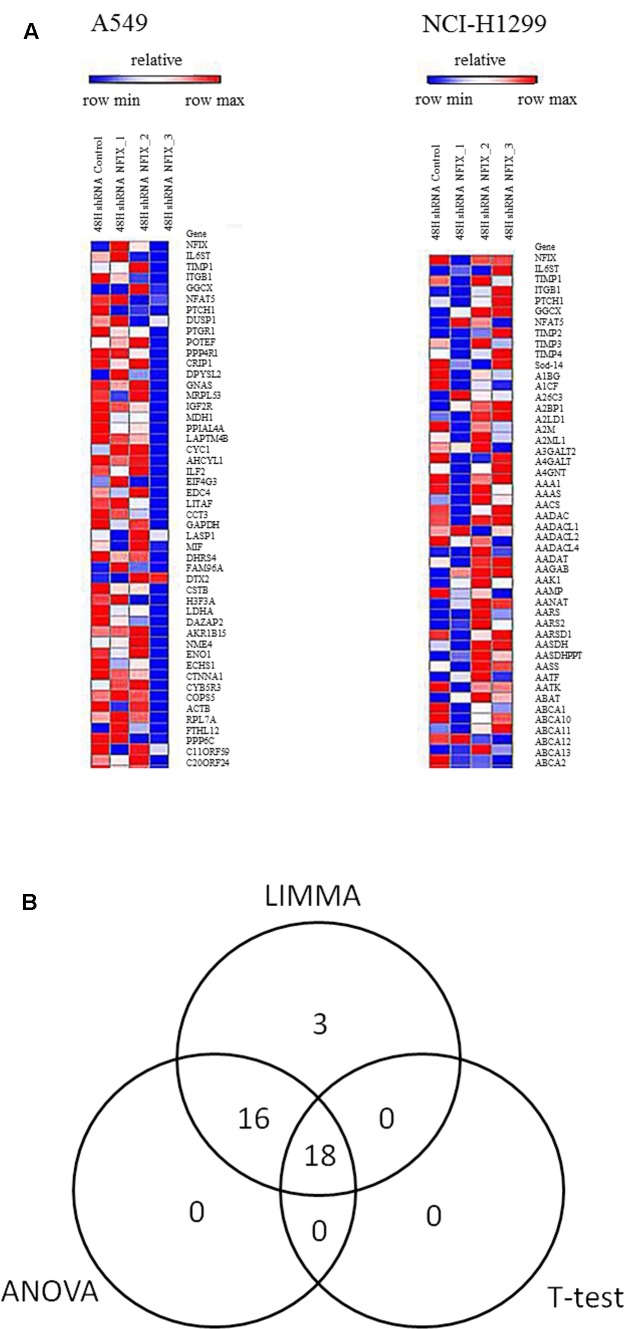
Heatmap from microarray gene expression analyses showing the differentially expressed genes in the A549 and NCI-H1299cell lines. **(A)** Shows differentially expressed genes in both cell lines at48h post–*NFIX* shRNAs transfection as compared to the non-silenced samples. **(B)** *T*-test, LIMMA and ANOVA were used to identify differentially expressed genes between *NFIX* silencing and non-silencing in the A549 and NCI-H1299cell lines. In total, 18 genes were identified as differentially expressed between both cell lines (^∗^with Benjamini-Hochberg adjustment with *p*-value < 0.05).

Gene expression microarray analyses were carried out to find differentially expressed genes before and after *NFIX* knockdown by comparing each cell line with respective controls at *p*-value < 0.05. **Figure 6A** depicts the heatmap of the top 50 genes including *NFIX*, *IL6ST*, *TIMP1* and *ITGB1*, for both cell lines at 48 h post-transfection. All differentially expressed genes in both cell lines from microarray analyses were integrated before undergo the statistical analyses whereas **Figure 6B** demonstrates the results of integrated analyses from all statistical software used. **Table 5** listed 18 genes identified via integrated analyses with respect to their fold change. All 18 genes were mapped into pathways to determine their involvement in lung cancer metastasis and how they are regulated by *NFIX*.

**Table 5:** The 18 differentially expressed genes in A549 and NCI-H1299 cell lines.

No.	Gene	Fold change (FC)	Log FC
1	*CHAC1*	3.26	1.70
2	*HIST1H2AC*	2.34	1.22
3	*MAP2*	2.13	1.09
4	*NFAT5*	1.94	0.95
5	*ZNF224*	1.92	0.94
6	*MAP2*	1.91	0.93
7	*CHD2*	1.86	0.89
8	*SNAP25*	1.78	0.83
9	*PTCH1*	1.45	0.54
10	*CLTB*	0.77	-0.38
11	*NUDT15*	0.76	-0.39
12	*POLR2K*	0.75	-0.41
13	*LAS1L*	0.73	-0.45
14	*CCT3*	0.70	-0.51
15	*C16orf53*	0.64	-0.64
16	*PAQR4*	0.63	-0.67
17	*SAC3D1*	0.58	-0.79
18	*STRA13*	0.56	-0.83


### Construction of a New Regulatory Pathway from Genes Identified in Microarray Analyses

**FIGURE 7:**
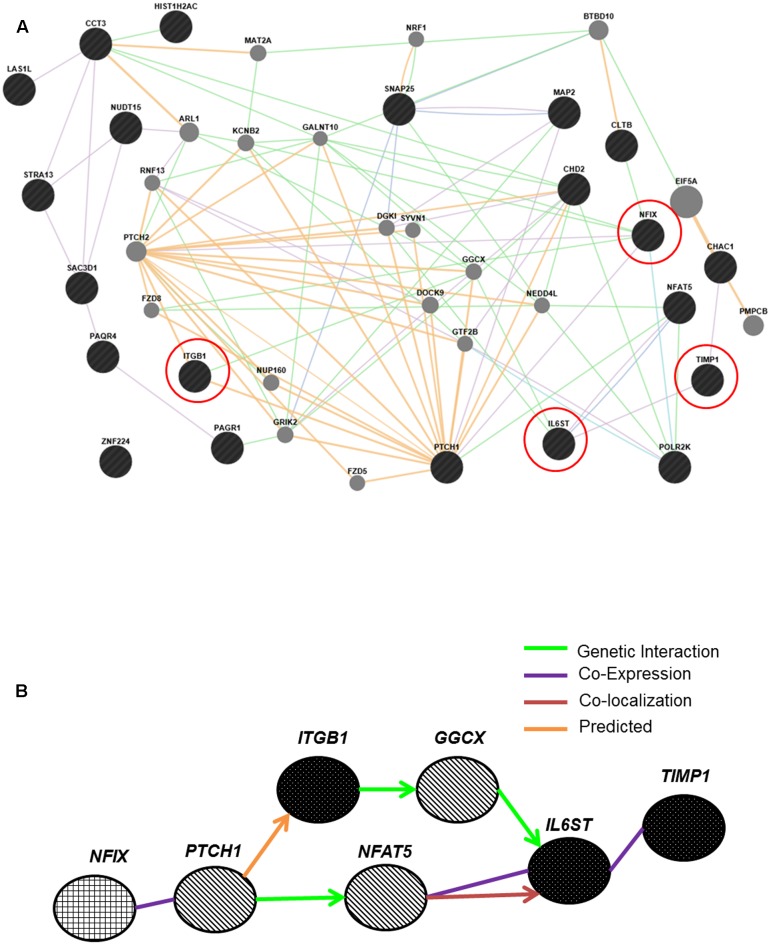
Pathways created by GeneMANIA connect the master regulator *NFIX* and its regulated genes *IL6ST*, *TIMP1* and *ITGB1.* **(A)** 17 differentially expressed genes from microarray analyses showed connection with all regulated genes and *NFIX*. Some genes showed co-expression with *NFIX*, including *PTCH1*. **(B)** Successful construction of a new transcriptional network from both meta-analysis and microarray analyses. *NFIX* was identified as a master regulator that regulates the *ITGB1*, *TIMP1* and *IL6ST* genes. Three new entities: the *PTCH1*, *NFAT5* and *GGCX*, were also included in this metastasis regulatory network for lung cancer. *NFIX* is the master regulator; *IL6ST*, *TIMP1* and *ITGB1* are the *NFIX*-regulated genes and *PTCH1*, *NFAT5* and *GGCX* are genes involved in the *NFIX* transcriptional regulatory network.

The regulated genes *ITGB1, TIMP1* and *IL6ST*, were included in the pathway altogether with 17 genes identified in the gene expression microarray using GeneMANIA. One pathway was selected as a new regulatory pathway network where it contained the most regulated genes in lung cancer metastasis as explained in **Figure 2** above. **Figure 7A** shows several new pathways created from genes identified in the meta-analysis and the microarray analyses. One direct network was selected prior to determination of their functions using GeneCards. *NFIX*, a master regulator regulates *ITGB1*, *TIMP1* and *IL6ST*, as well as three new entities, *PTCH1*, *NFAT5* and *GGCX*, were included in this new transcriptional network as shown in **Figure 7B**.

## Discussion

The understanding of tumor progression and TFs are critical in the efforts of identifying new biomarkers, inventing novel therapeutics, making patients’ prognosis and designing an advance cancer detection tools (Gomez, 2016). The identification of gene signatures for metastasis is crucial for preventing tumor cells from metastasizing, to improve lung cancer prognosis as well as increased survival. The target gene could be used for future therapy to inhibit the metastasis process in lung cancer. The identification of TFs that regulate gene expression is essential for understanding the whole transcription process, particularly the genes involved in metastasis. In this study, we identified 17 genes involved in the migration pathway using DAVID and Pathway Studio. Subsequently, 10 kb upstream of the promoter sequences of these 17 genes were retrieved and finally we postulated that *NFIX* is a master regulator for lung cancer metastasis. We showed evidence that *NFIX* regulates *IL6ST*, *TIMP1* and *ITGB1* genes. Altogether, these genes promote cell migration and invasion, which lead to lung cancer aggressiveness, poor survival and high mortality rates. However, further study need to be done to understand the molecular mechanism of *NFIX* in regulating these regulated genes and functional pathways of metastasis.

In addition to *NFIX*, *AP1* was also identified more occurrences at 10 kb upstream of the promoter sequence. The *AP1* gene is well studied in the migration pathway of breast cancer via the extracellular signal–regulated kinase pathway (Chen et al., 2009). It plays an important role in regulating endothelin-1 (*ET1*) gene transcription in endothelial cells (Kawana et al., 1995). The TF *SP1* also identified with the highest occurrences; it is highly expressed in the tumor cells progression of gastric cancer (Wang et al., 2003). Blomquist et al. (1999) showed that *NFIX* acts as a specific dimer to DNA sequences with high affinity which can increase the specificity for molecular recognition and aid targeted therapy in lung cancer (Blomquist et al., 1999).

Silencing of *NFIX* reduced the expression of its regulated genes *IL6ST*, *TIMP1* and *ITGB1*. Necula et al. (2012) showed that increased levels of *IL6ST* in tissues (>758 pg/ml) and plasma (>38.21 pg/ml) are associated with shorter survival in gastric adenocarcinoma. *IL6* manipulates the tumorigenesis process by activating the genes involved in differentiation, survival, apoptosis and proliferation (Lin et al., 2007). In addition, *IL6* may be involved in promoting inflammation by inducing anti-apoptotic signals which is mediated by *STAT3* (Necula et al., 2012). Lissat et al. (2015) showed that high expression of *IL6ST* aids Ewing sarcoma tumor progression and renders it resistant to apoptosis and promotes metastasis, thus suggesting that *IL6ST* plays a role in cancer metastasis. *TIMP1* promotes melanoma through activation of the AKT pathway independent of the PI3K signaling pathway (Toricelli et al., 2013). In human melanoma cell lines, *TIMP* interacts with *ITGB1* and *CD63* and assists melanomagenesis and resistance to the cell death program (Toricelli et al., 2013).

A previous study of *ITGB1* demonstrated its association with migration activity, invasion and wound healing in an *in vitro* experiment of lung adenocarcinoma (Wang et al., 2013). The authors reported that *osteopontin*, *LAMB3* and *ITGB1* are pro-metastatic genes for lung cancer. *Osteopontin* also leads to increased vascular endothelial cell migration, proliferation, angiogenesis and tumor growth in lung cancer (Cui et al., 2007; Fong et al., 2009). Lymphatic metastasis in lung cancer has a high concentration of *ITGB1* as compared to non-lymphatic metastasis (Wang et al., 2013). Our data demonstrate similar results in the *in vitro* experiments, where significant reduction of cell proliferation, migration and invasion was observed post–*NFIX* knockdown. The *in vitro* studies were in concordance with the *in silico* analyses predictions, where *NFIX* regulates *IL6ST*, *TIMP1* and *ITGB1* expression.

Microarray analyses were carried out to discover differentially expressed genes in A549 and NCI-H1299 cell lines post–*NFIX* silencing as compared to non-silenced cells. Silencing of cell line A549 using NFIX_3 shRNA does not show any difference compared to the other shRNAs. We suggest that the sequence in NFIX_1 shRNA is the most powerful in silencing the master regulator *NFIX* especially in aggressive cell line NCI-H1299. *NFAT5* was identified as a new entity in this network. *NFAT5* is a crucial component in tumor development and progression, particularly in regulating inflammation in the carcinogenesis process (Neuhofer, 2010; Yoon et al., 2011). We also observed that *PTCH1* is co-expressed with *NFIX. PTCH1* serves as a tumor suppressor gene as demonstrated in NSCLC cell lines (Shikata et al., 2011). Li et al. (2012) showed that *PTCH1* interacts with the Hedgehog (Hh) signaling pathway via the effect of miRNA-212 on cell proliferation. However, our microarray data showed that *PTCH1* is co-expressed with *NFIX*. Further studies on the co-expression between these two genes need to be performed in the future. The *GGCX* gene, encoding the enzyme responsible for the post-translational modification of vitamin K–dependent proteins in haemostasis (Stafford, 2005; Weston and Monahan, 2008), was also found in our newly constructed transcriptional regulatory network. Our findings suggest that *NFIX* may be a potential biomarker with high potential for inhibiting the metastatic process in NSCLC as demonstrated by the *in-silico* and *in vitro* experiments.

## Conclusion

*In silico* analyses identified *NFIX* as a predicted master regulator. Based on the *in silico* findings, functional assays and microarray, a new transcriptional network involving the inflammation, migration and invasion pathways specific to metastatic lung cancer was created. We acknowledged the *in-silico* results are considered the limitation of our study as the results of the same analyses will always be different with the new additions from new discoveries as the programmer might update and upgrade the software and algorithms. We used several *in silico* analyses to strengthen and support our findings. We also ran all *NFIX* shRNAs lentiviral with different sequences in our study to check the best shRNA needed to significantly reduce the expression of the master regulator and gene functions that lead to metastasis phenotypic outcome. We suggest that *NFIX* is a master regulator that regulates metastasis in lung cancer by transcribing the genes responsible in activating the pathways leading to micrometastasis and finally cause the cancer cells to metastasize such as inflammation, proliferation, migration and invasion. Where its’ silencing can reduce cellular proliferation, migration and invasion *in vitro* especially as observed in aggressive cell line. Hence, *NFIX* with *IL6ST*, *TIMP1*, *ITGB1*, *PTCH1*, *GGCX* and *NFAT5* genes may regulate the migration and invasion process and they could serve as potential therapeutic targets in patients with lung cancer to predict survival and improve prognosis. We couldn’t carry the *in vivo* and also protein study experiments due to lack of funding. However, more detailed functional studies, *in vivo* and clinical validation need to be carried out in the future to prove this concept and make the study more transparent and reliable.

## Author Contributions

This study was planned by RH. All experiments were carried out by NIAR as described in the manuscript including interpretation of data and statistical analyses. MMM participated in microarray data analysis. RH and NAAM supervised the student, assisted in study and manuscript writing. The manuscript was written by NIAR with comments from all co-authors and mostly from NAAM, RH, and RJ. All co-authors read and approved the final version of the manuscript.

## Conflict of Interest Statement

The authors declare that the research was conducted in the absence of any commercial or financial relationships that could be construed as a potential conflict of interest.

## Footnotes

1 www.oncomine.org/

2 http://epd.vital-it.ch/

3 http://www.ncbi.nlm.nih.gov/

4 http://alggen.lsi.upc.es/cgi-bin/promo_v3/promo/promoinit.cgi?dirDB=TF_8.3
